# Preventive Effect of Gonggan (Citrus Reticulata Blanco Var. Gonggan) Peel Extract on Ethanol/HCl-Induced Gastric Injury in Mice *via* an Anti-oxidative Mechanism

**DOI:** 10.3389/fphar.2021.715306

**Published:** 2021-11-17

**Authors:** Ya Wu, Hua Jiang, Guangfang Chen, Xingxing Chen, Chengming Hu, Xiaofei Su, Fang Tan, Xin Zhao

**Affiliations:** ^1^ Chongqing Collaborative Innovation Center for Functional Food, Chongqing University of Education, Chongqing, China; ^2^ Chongqing Engineering Research Center of Functional Food, Chongqing University of Education, Chongqing, China; ^3^ Chongqing Engineering Laboratory for Research and Development of Functional Food, Chongqing University of Education, Chongqing, China; ^4^ College of Biological and Chemical Engineering, Chongqing University of Education, Chongqing, China; ^5^ Department of TCM (Rheumatic Immunology/Geriatrics), People’s Hospital of Chongqing Banan District, Chongqing, China; ^6^ Department of Public Health, Our Lady of Fatima University, Valenzuela, Philippines

**Keywords:** citrus peel, gastric injury, anti-oxidative, anti-inflammation, mice

## Abstract

Gonggan (Citrus reticulata Blanco var. gonggan) is one of the most popular citruses. In this study, the effect of Gonggan peel extract (GPE) on gastric injury was investigated. The components in GPE were analysed by HPLC and the gastric injury model in mice was established by ethanol/hydrochloric acid. After treatment by GPE, the pathological changes of gastric tissue were observed by optical microscope. The levels of oxidative stress and inflammation were measure by kit. And the mRNA expression of related gene was determined by qPCR assay. HPLC result showed GPE mainly contained the flavonoids narirutin, hesperidin, nobiletin, tangeretin and 5-demethylnobiletin. Morphological and pathological analysis of gastric tissue revealed that GPE could relieve gastric injury. Also, GPE increased the levels of SOD, GSH-Px, and CAT and decreased the level of MDA. Moreover, GPE decreased the levels of the inflammatory cytokines TNF-α, IFN-γ, IL-1β, and IL-6 to suppress inflammation. In addition, the q-PCR results showed that GPE upregulated the mRNA expression of SOD1, SOD2, γ-GCS, GSH-Px, CAT, and IκBα and downregulated the mRNA expression of NF-κB. In conclusion, GPE alleviated gastric injury caused by ethanol/hydrochloric acid by inhibiting oxidative stress and the inflammatory response. The mechanism by which GPE protects gastric tissues may involve the antioxidative pathway. Therefore, GPE has great potential to be developed as a product to prevent gastric injury.

## Introduction

Citrus is the largest category of fruit in the world, which has a long history of cultivation in China (Liu et al., 2012). Moreover, citrus has high healthcare, medical and economic value (Liu et al., 2012). The peel of citrus fruit contains a variety of bioactive substances, such as essential oil and flavonoids (Gao et al., 2018; Tripoli et al., 2007; Asikin et al., 2012). Pharmacological studies have shown that bioactive compounds in citrus peel are active ingredients, with antioxidant (Zou et al., 2016), anti-inflammatory (Chen et al., 2017; Gabriele et al., 2017), lipid metabolism-regulating (Toth et al., 2016), anticancer (Ke et al., 2015), neuroprotective and other biological activities (Cirmi et al., 2016). The Gonggan (Citrus reticulata Blanco var. gongan) is abundant in Guangdong Province of China. The annual output of it was over 320,000 tons (Cai et al., 2021). And Gonggan is rich in nutrition and unique in taste, so it is popular among the people. After Gonggan is picked, a large amount of by-products, such as peel, fruit seeds, will be produced. There are few researches on the application and activity of flavonoids in Gonggan peel, only the study of its antibacterial effect was reported (Deng and Chao, 2013). Therefore, related research about the utilization of active ingredients in peel residue needs to be explored, which provides an opportunity for the development of citrus industry and human health (Ledesmaescobar and Castro, 2014).

With the development of economy, alcoholic beverages have become one of the main beverages in people’s daily life, especially in occasions such as weddings and parties. The total alcohol consumption per capita increased from 5.5 L in 2005 to 6.4 L in 2016 in the population aged over 15 years (World Health Organization, 2019). Excessive drinking is a factor leading to more than 200 diseases and injuries (World Health Organization, 2019). Thus, it is of great significance to study the related diseases caused by alcohol. Gastric injury is a common clinical disease, and alcohol is an important factor that induces gastric injury (Qin et al., 2019). Ethanol is the main component of liquor and alcoholic beverages. High concentrations of ethanol can not only directly erode gastric mucosal tissues, but also cause the production and release of many free radicals and inflammatory mediators, which leads to gastric injury (Zhang et al., 2006; Raish et al., 2018). As a common solvent, ethanol residues are strictly limited in food and pharmaceutical products. Flavonoid monomers have good anti-inflammatory and antioxidant properties. Citrus peels are rich in flavonoids, but there are few studies on the physiological activities of citrus peel extract in gastric injury models. Based on gastric injury caused by alcohol is a common phenomenon, the study of citrus peel extract to protect against gastric injury not only helps to promote the research of gastric diseases, but also makes full use of the waste of citrus peel.

In this study, we used the Gonggan peel extract (GPE) and established acute gastric injury in mice induced by ethanol/hydrochloric acid to evaluate the effect of GPE. The mouse gastric lesion area, pathological changes, biochemical indicators, and cytokines, were examined. Moreover, the expression of related genes was measured by q-PCR to elucidate the possible mechanism of GPE. This study provides experimental support for the utilization and exploitation of citrus peel.

## Materials and Methods

### GPE Preparation

Gonggan (Citrus reticulata Blanco var. gonggan from Yulin City, Guangxi Zhuang Autonomous Region, China) peel was removed and crushed into powder after being freeze-dried in lyophilizer (Ningbo Scientz Biotechenology Co., Ltd. Ningbo City, China). One hundred grams of dried citrus peel powder was added to 80% ethanol with a liquid-to-material ratio of 20:1 and then heated at 80°C for 4 h (Sharma et al., 2019). AB-8 macroreticular resin (Beijing Solarbio Science and Technology Co., Ltd. Beijing, China) was used to purify the crude extract. The macroporous adsorption resin was eluted by 90% ethanol (5 BV) and the eluent rate was 15 ml/min (Nie et al., 2017). Ethanol eluent was combined and evaporated under reduced pressure. The residue was freeze-dried and then ground to produce powder of GPE. The total flavonoids of GPE were obtained by detecting the absorbance at the wavelength of 500 nm (Evolution 300 ultraviolet spectrophotometer, Thermo Fisher Scientific, Inc. Waltham, MA, United States) with rutin as the standard substance according to the literature (Liu et al., 2019).

### Determination of GPE Composition

We accurately weighed 2 mg of each of the following: narirutin, hesperidin, nobiletin, tangeretin, and 5-demethylnobiletin (Shanghai Yuanye Biological Technology Co., Ltd. China). Then, the standard substances were dissolved in methanol (HPLC grade) individually to afford standard solutions of different concentrations. Then, 5 mg of GPE was dissolved in methanol (1 ml). This sample solution was filtered through a microporous membrane (0.22 μm) to afford the test solution.

The chromatographic separation conditions were carried out as follows: a liquid chromatography system (UltiMate3000 HPLC System, Thermo Fisher Scientific, Waltham, MA, United States) with a Welch C18 column (4.6 × 250 mm long, 5 μm) was used, mobile phase A was acetonitrile (HPLC grade), and mobile phase B was 0.5% glacial acetic acid aqueous solution. The mobile phase gradient was as follows: 0 min, 12% A; 0–20 min 25% A; 20–35 min 45% A; 35–40 min, 100% A. The flow rate was set at 0.5 ml/min, and the column temperature was 35°C. The injection volume was 5 μl, and the detection wavelength was 285 nm. The sample solution and standard solutions of different concentrations were analysed under these conditions. The compounds in GPE were analysed according to the chromatographic peak area of the standard substance.

### Determination of Antioxidant Capacity *in Vitro*

The antioxidant capacity of GPE *in vitro* was studied. GPE was dissolved in absolute ethanol to prepare gradient concentration of test solution (0.1–0.8 mg/ml). The antioxidant capacity of GPE was evaluated by measuring 2,2′-diphenyl-1-picrylhydrazyl radical (DPPH˙), 2, 2′-azino-bis (3-ethylbenzothiazoline-6-sulfonic acid) radical cation (ABTS˙^+^), hydroxyl radical (HO˙) scavenging activity following the method previously reported (Kavoosi and Amirghofran, 2017).

### Animal Models and Treatments

Fifty specific pathogen-free (SPF) Kunming mice aged 6 weeks (20 ± 2 g, male, from Chongqing Medical University, Chongqing, China) were kept at 25°C with humidity of 50 ± 5%, alternating between day and night for 12 h. After adaptive feeding for a week, mice were divided into five groups with 10 mice in each group: the normal group (control), model group, the low-concentration GPE group (GPE-L group), the high-concentration GPE group (GPE-H group), and the ranitidine group. Ranitidine could reduce gastric acid and pepsin activity, which is often used as a positive agent in gastric injury (Liu et al., 2019). All mice in the normal group were fed a normal maintenance diet and drank water. The GPE powder was dispersed in 0.5% carboxymethylcellulose sodium aqueous solution to prepare GPE solution for gavage. Mice in the GPE-H group and GPE-Lgroup were intragastrically administered 300 (6 mg/ml) and 150 (3 mg/ml) mg/kg GPE daily, respectively. Mice in the ranitidine group were intragastrically administered 50 mg/kg ranitidine daily. The mice in model and normal group were intragastrically administered same volume of 0.5% carboxymethylcellulose sodium aqueous solution. After 14 days drug treatment (before established gastric injury model), the average weight of each group of mice is about 38 g. And the mice with smooth fur move normally and react quickly. On the 15th day, all mice were fasted for 24 h, and the mice, except those in the normal group, were intragastrically administered a solution of anhydrous ethanol and 15% hydrochloric acid (volume ratio = 4:6, 0.1 ml/10 g) (Zhao et al., 2013; Suo et al., 2016). After 0.5 h, mice were anesthetized with ether, and then blood was collected from the orbital sinus. Subsequently, the mice were sacrificed by cervical dislocation. Blood was centrifugated at 3,000 rpm/min for 10 min, the supernatant was collected and stored at −80°C for further serological analysis. The gastric tissue was cut along the great curve of the stomach and photographed. Formula (1 - gastric injury area of sample treated mice/gastric injury area of injured group mice × 100%) was used to calculate the inhibitory rate of gastric injury. Part of the gastric tissue was resected for histopathological examination. The remaining tissues were stored at −80°C for further analysis.

### Histological Analysis of Gastric Tissue

After fixation in 10% formalin solution for 48−h, gastric tissue (∼0.5 cm^2^) was dehydrated, embedded in paraffin, sectioned, and stained with hematoxylin and eosin. An optical microscope was used to examine the pathological changes in the gastric tissue (BX43 microscope, Olympus, Tokyo, Japan). Histological score of gastric tissues was evaluated according to the method previously reported (Amirshahrokhi and Khalili, 2015): 1) mucosal edema (score 0–4), 2) hemorrhage (score 0–4), 3) inflammatory cell infiltration (score 0–3), and 4) epithelial cell loss (score 0–3).

### Determination of SOD, CAT, GSH-Px and MDA Levels in Gastric Tissue

A 10% tissue homogenate was obtained by grinding the gastric tissue in a 0.9% sodium chloride solution. After centrifugation at 10,000 rpm/min for 15 min, the supernatant of the 10% tissue homogenate was collected for analysis. The levels of SOD (catalog number: A001-1–2), GSH-Px (catalog number: A005-1–2), CAT (catalog number: A007-1-1) and MDA (catalog number: A003-1–2) in gastric tissue were analysed by using corresponding test kits (Nanjing Jiancheng Bioengineering Institute, Nanjing, Jiangsu, China). The tissue homogenate was processed according to the kit instructions, and the absorbance was measured at the required wavelength in Varioskan LUX Multimode Microplate Reader Fluoroskan (Thermo Fisher Scientific, Waltham, MA, United States).

### Determination of EGF, SS, TNF-α, IFN-γ, IL-1β, and IL-6 in Serum

After the serum thawed, the serum levels of EGF (catalog number: H031), SS (catalog number: H092), TNF-α (catalog number: H052-1), IFN-γ (catalog number: H025), IL-1β (catalog number: H002), and IL-6 (catalog number: H007-1-1) were measured by Mouse ELISA Kit (Nanjing Jiancheng Bioengineering Institute, Nanjing, Jiangsu, China). After treating the serum according to the kit instructions, the absorbance was measured at 450 nm in Varioskan LUX Multimode Microplate Reader Fluoroskan (Thermo Fisher Scientific, Waltham, MA, United States) to calculate the content.

### Quantitative PCR

The gastric tissue (50 mg) was minced in 1 ml Trizol reagent (Invitrogen, Carlsbad, CA, United States). The homogenate was added to 200 μl of trichloromethane and the mixture was left on ice for 5 min. After centrifuged at 14,000 rpm/min for 15 min, the supernatant was added with equal volume of isopropanol. The solution was centrifuged at 14,000 rpm/min for 15 min. Then, the supernatant was discarded and the precipitation was cleaned with the solution of enzyme free water and ethanol (volume ratio = 1:3). After centrifuged at 14,000 rpm/min for 15 min, residue was dissolved in 20 μl enzyme free water to afford the total RNA. The purity and concentration of the total RNA were tested *via* ultra-microspectrophotometry (Nano-100, All for Life Science, Hangzhou, Zhejiang, China), The RNA was diluted to a concentration of 1 μg/μl with enzyme free water. Reverse transcription kit (Tiangen Biotech Co., Ltd., Beijing, China) was used to synthesize cDNA template. Oligo (dT) primer (9 μl) was added into the solution of diluted RNA (1 μ) and enzyme free water (10 μl), then the mixed solution was put into the polymerase chain reaction (PCR) equipment at 65°C for 5 min. After cooling, the reaction system was added with 4 μl of 5× reaction buffer, 1 μl of RiboLock RNase inhibitor, 2 μl of 10 mM dNTP mix, and 1 μl of RevertAid Reverse transcriptase. The cDNA template was synthesized in PCR equipment at 42°C for 60 min and 70°C for 5 min. A solution of SYBR Green PCR Master Mix (10 μl) and cDNA template (1 μl) was added to the upstream and downstream primers (Table 1, 1 μl) to prepare the qPCR reaction system. The reaction was carried out at Applied Biosystems StepOnePlus ™ Real-Time PCR Instrument (Thermo Fisher Scientific Co., Ltd., Massachusetts, United States) as follows: 95°C for 90 s, 40 cycles of 95°C for 30 s, 60°C for 30 s, 72°C for 30 s, then 95°C for 30 s, and 55°C for 35 s. The 2^−ΔΔCt^ method was used to calculate relative gene expression, and glyceraldehyde-3-phosphate dehydrogenase (*GAPDH*) was used as a housekeeping gene.

**TABLE 1:** Sequences of primers used in this study.

Gene name	Sequence
*SOD1*	Forward: 5′-AAC​CAG​TTG​TGT​TGT​CAG​GAC-3′
	Reverse: 5′-CCA​CCA​TGT​TTC​TTA​GAG​TGA​GG-3′
*SOD2*	Forward: 5′-CAG​ACC​TGC​CTT​ACG​ACT​ATG​G-3′
	Reverse: 5′-CTC​GGT​GGC​GTT​GAG​ATT​GTT-3′
*CAT*	Forward: 5′-AGC​GAC​CAG​ATG​AAG​CAG​TG-3′
	Reverse: 5′-TCC​GCT​CTC​TGT​CAA​AGT​GTG-3′
γ-GCS	Forward: 5′-CTT​CCC​TCC​CTT​CGG​ATC​G-3′
	Reverse: 5′-GTC​CAC​AGA​GAT​GCA​GTG​AAA-3′
*GSH-Px*	Forward: 5′-AAT​GTC​GCG​TCT​CTC​TGA​GG-3′
	Reverse: 5′-TCC​GAA​CTG​ATT​GCA​CGG​G-3′
*NF-κB p65*	Forward: 5′-TGC​GAT​TCC​GCT​ATA​AAT​GCG-3′
	Reverse: 5′-ACA​AGT​TCA​TGT​GGA​TGA​GGC-3′
*IκBα*	Forward: 5′-TGA​AGG​ACG​AGG​AGT​ACG​AGC-3′
	Reverse: 5′-TGC​AGG​AAC​GAG​TCT​CCG​T-3′
*GAPDH*	Forward: 5′-TGG​CCT​TCC​GTG​TTC​CTA​C-3′
	Reverse: 5′-GAG​TTG​CTG​TTG​AAG​TCG​CA-3′

### Statistical Analysis

The experimental data were analysed with SPSS 17.0 (SPSS Inc. Chicago, IL, United States) and GraphPad Prism seven statistical software (GraphPad Software Inc. La Jolla, CA, United States). The results are expressed as the mean ± standard deviation. Comparisons among groups were made by Kruskal-Wallis ANOVA, post hoc Dunn’s multiple comparison test (non-parametric tests). A value of *p* ≤ 0.05 indicated that the difference was statistically significant.

## Results

### Composition Analysis of GPE

**FIGURE 1:**
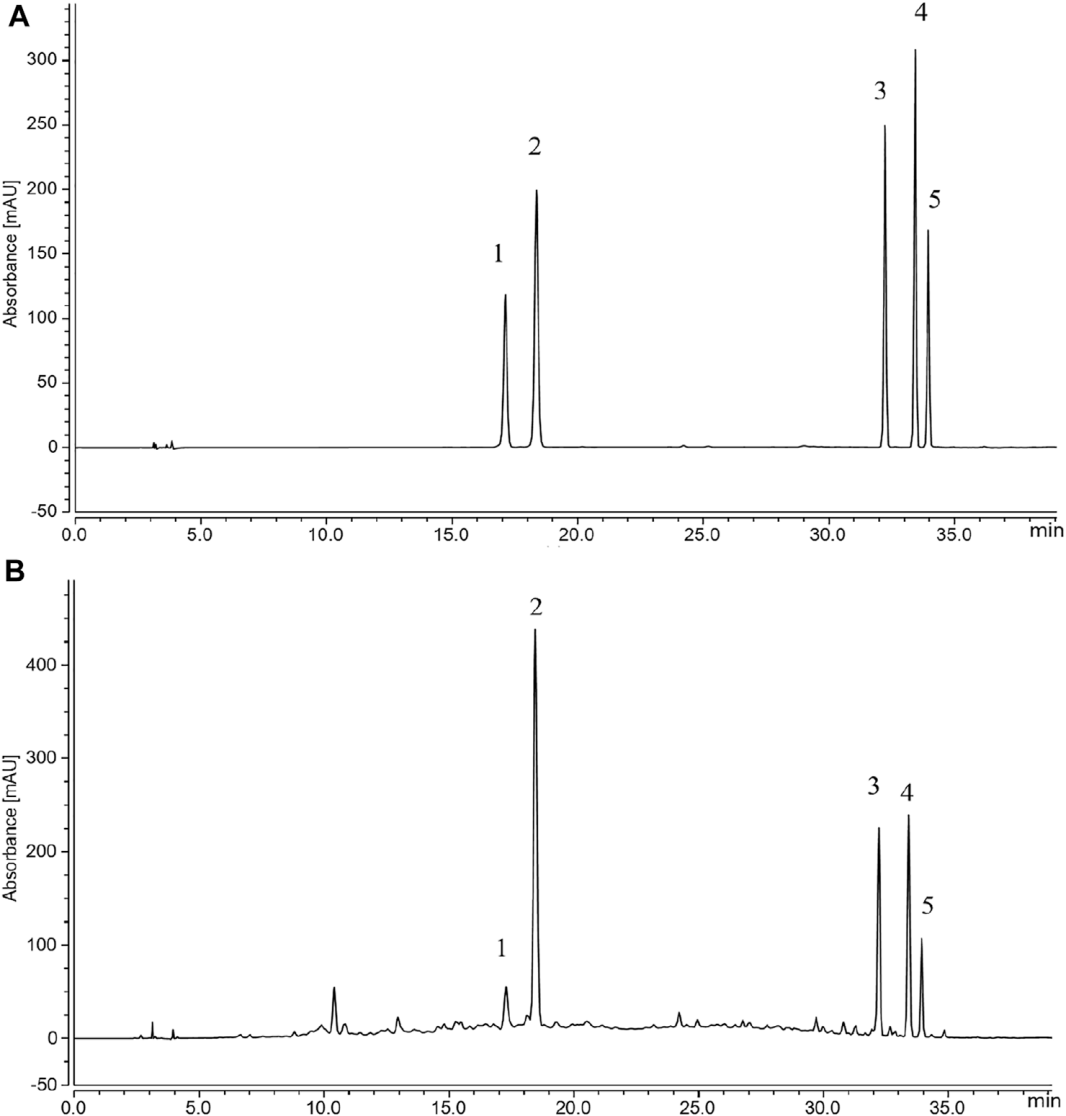
Flavonoids in GPE **(A)** Standard chromatograms; **(B)** Flavonoid constituents of GPE: 1. narirutin; 2. hesperidin; 3. nobiletin; 4. tangeretin; 5.5-demethylnobiletin.

The total flavonoids in GPE were determined to be 11.7%. The regression equations of flavonoid standards were shown in Table 2, where x is the concentration of flavonoids and the y is the peak area tested by HPLC. The HPLC results (Figure 1) showed the flavonoids monomer in GPE as follows: narirutin (11.9 mg/g), hesperidin (84.9 mg/g), nobiletin (24 mg/g), tangeretin (27.2 mg/g), and 5-demethylnobiletin (9.9 mg/g).

**TABLE 2:** The regression equations of flavonoid standards.

Standards	concentration range (mg/ml)	egression equation	R^2^
narirutin	0.03125–0.2500	y = 153.16x-0.7348	0.9856
hesperidin	0.05415–0.4332	y = 156.71x**-**0.8329	0.9949
nobiletin	0.0375–0.3000	y = 184.55x**+**0.4242	0.9998
tangeretin	0.04688–0.3750	y = 171.33x**+**0.5235	0.9999
5-demethylnobiletin	0.01719–0.1375	y = 248.77x**+**0.1674	0.9999

### The Scavenging Activity of GPE

**FIGURE 2:**
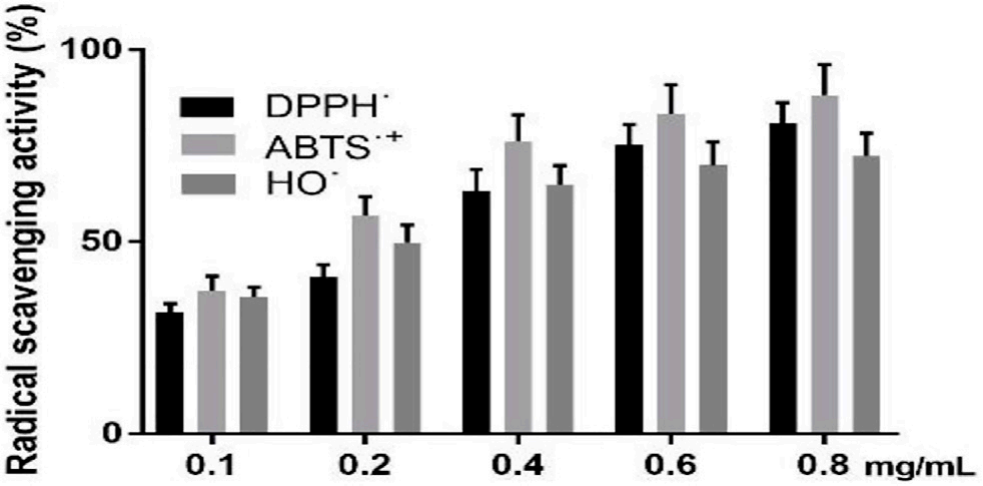
Radical scavenging activity of DPPH˙, ABTS˙^+^ and OH˙. Data are shown as means ± SD (n = 3).

Figure 2 shows that as the concentration of GPE was increased, the ability to scavenge free radicals was gradually increased. When the GPE concentration is 0.8 mg/ml, the scavenging rates of DPPH˙, ABTS˙^+^ and OH˙ are 80.8,88.2, and 72.2%, respectively.

### Inhibition of Gastric Injury

**FIGURE 3:**
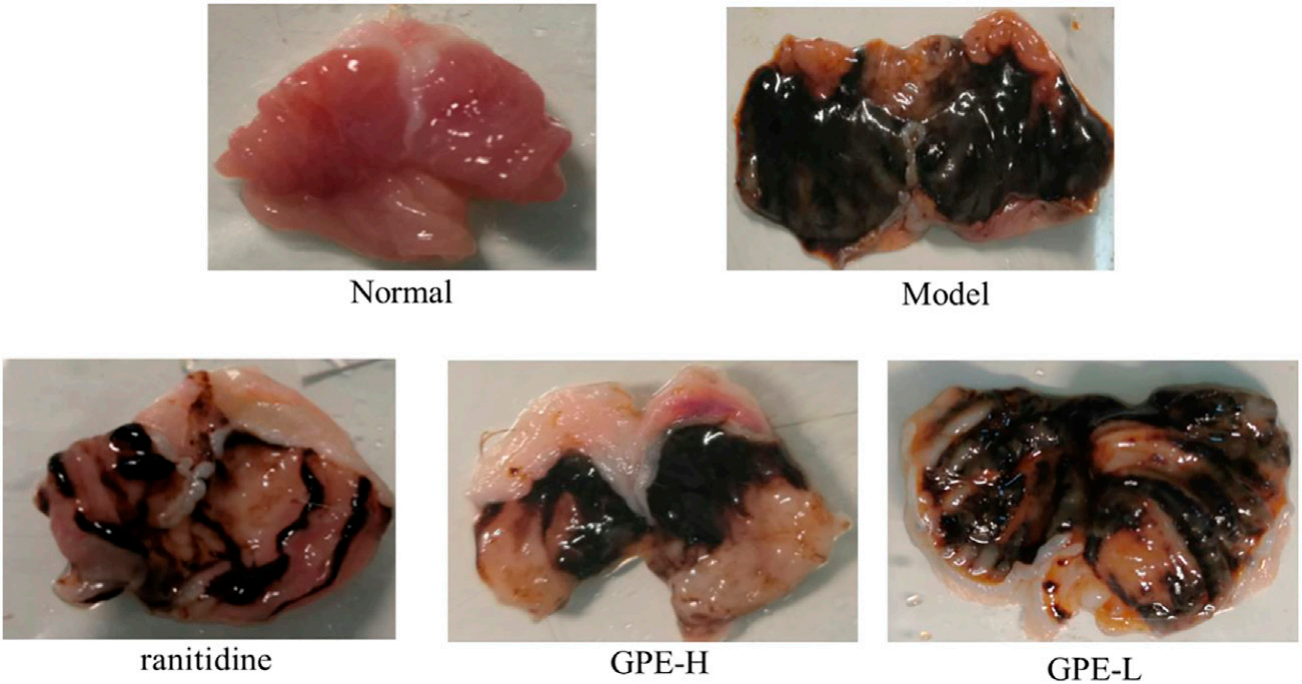
Images of stomach specimens from mice of each group. ranitidine, mice treated with 50 mg/kg ranitidine; GPE-H, mice treated with 300 mg/kg GPE; GPE-L, mice treated with 150 mg/kg GPE.

After treatment with ethanol/HCl solution, the gastric mucosa was severely damaged in the model group (Figure 3). Compared with the model group, the gastric injury of treatment group was relieved to varying degrees. It was obvious that the damage in the GPE-L group was more serious than that in the GPE-H and ranitidine groups. By calculating the gastric injury area with ImageJ software, the inhibition of gastric injury in each group could be directly compared. As shown in Table 3, the area of gastric injury in the model group was largest, which was significantly different from that of GPE-H and ranitidine group (*p* < 0.05). The inhibition rates of gastric injury in the treatment groups were increased to different degrees. There were no significant differences between the treatment groups. However, inhibition rates of gastric injury in GPE-H and ranitidine groups were more similar to that in the normal group.

**TABLE 3:** The gastric injury area and gastric injury inhibitory rate of mice.

Group	Gastric injury area (mm^2^)	Gastric injury inhibitory rate (%)
Normal	0.00 ± 0.00^a^	100 ± 0.0^a^
Model	180.6 ± 15.3^b^	0 ± 0.0^b^
ranitidine	40.1 ± 5.9^ac^	77.8% ± 3.3%^ac^
GPE-H	59.7 ± 6.1^ac^	67.2% ± 4.3%^ac^
GPE-L	82.5 ± 7.7^bc^	54.6% ± 6.4%^bc^

Values are presented as the mean ± standard deviation (n = 8/group). ranitidine, mice treated with 50 mg/kg ranitidine; GPE-H, mice treated with 300 mg/kg GPE; GPE-L, mice treated with 150 mg/kg GPE. ^a–d^ Mean values with different letters in the same column are significantly different (*p* < 0.05).

### Histopathological Examination of Gastric Tissue

**FIGURE 4:**
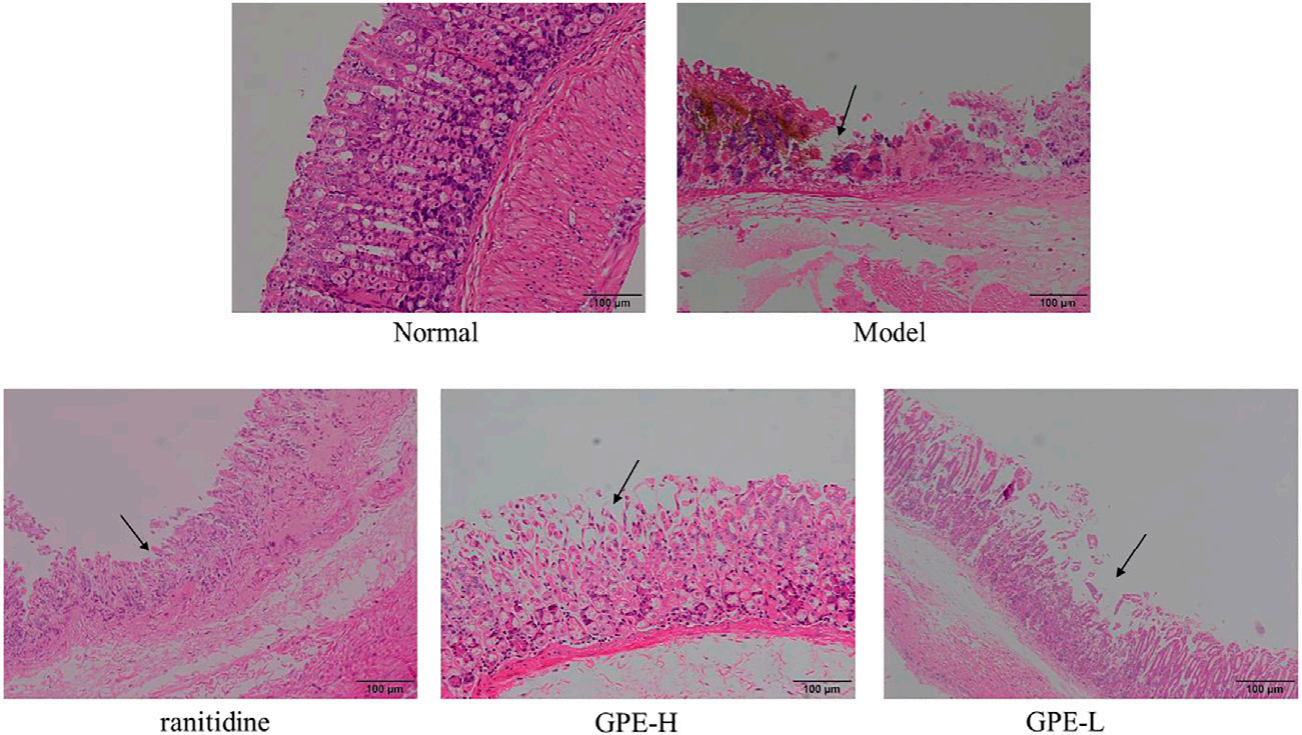
Histopathological observation of gastric tissues sections in mice of the different groups after staining with hematoxylin and eosin (H&E).

As shown in Figure 4, the structure of the epithelium was complete and continuous, and the glandular structure was arranged in order. In the model group, the surface structure of the gastric mucosa was seriously damaged, which had a disordered arrangement of glands. After treatment with GPE and ranitidine, the damage to gastric tissues was alleviated to a certain extent. Epithelial cells exhibited less necrosis, and the arrangement of glands was more orderly than that in the model group. The protective effect of GPE was dose-dependent. The results showed that GPE protected the gastric mucosa from injury induced by ethanol/hydrochloric acid to a certain extent. As shown in Table 4, compared with the model group, the injury degree of epithelial cells in treatment group was effectively alleviated, while no significant changes of hemorrhage and cell infiltration were observed in treatment groups.

**TABLE 4:** The pathological changes in gastric mucosa induced by ethanol/HCl in mice.

Group	Edema (score 0–4)	Hemorrhage (score 0–4)	Cell infiltration (score 0–3)>	Epithelial cell loss (score 0–3)
Normal	0	0	0	0
Model	2 (1–3)	2 (1–3)	1 (0–2)	3
ranitidine	1 (0–2)	0	0	1 (1–2)
GPE-H	1 (0–2)	0	0	1 (1–2)
GPE-L	1 (0–2)	0	0	2 (2–3)

Data are presented as median values with minimum and maximum values in parentheses. Ranitidine, mice treated with 50 mg/kg ranitidine; GPE-H, mice treated with 300 mg/kg GPE; GPE-L, mice treated with 150 mg/kg GPE.

### SOD, CAT, GSH-Px, and MDA Levels in Gastric Tissue

**FIGURE 5:**
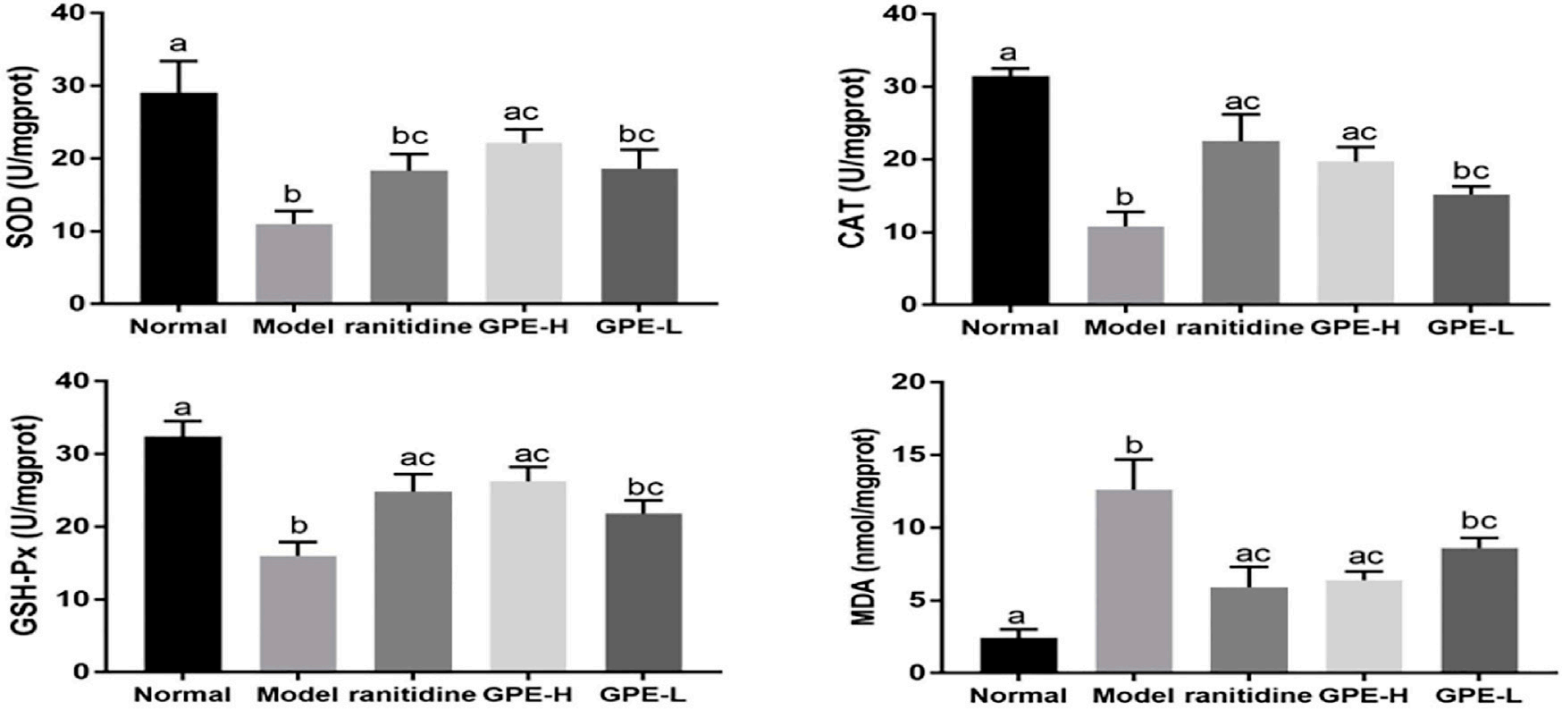
Gastric tissue levels of SOD, CAT, GSH-Px and MDA in mice. The data are shown as mean ± SD (n = 8). ^a–c^ Mean values with different letters are significant difference (*p* < 0.05) according to analysis of variance.

Compared to the normal group, ethanol/hydrochloric acid reduced the levels of SOD, CAT, and GSH-Px and elevated the level of MDA in the gastric tissue of the model group (Figure 5, *p* < 0.05). After treatment with GPE and ranitidine, the overall levels in the GPE-H and ranitidine groups were close to those in the normal group. The SOD level of ranitidine group was not significantly different from that of model group (*p* > 0.05), indicating that effect of ranitidine on SOD improvement was poor.

### Serum EGF, SS, TNF-α, IL-6, IL-1β, and IFN-γ Levels in Mice

**FIGURE 6:**
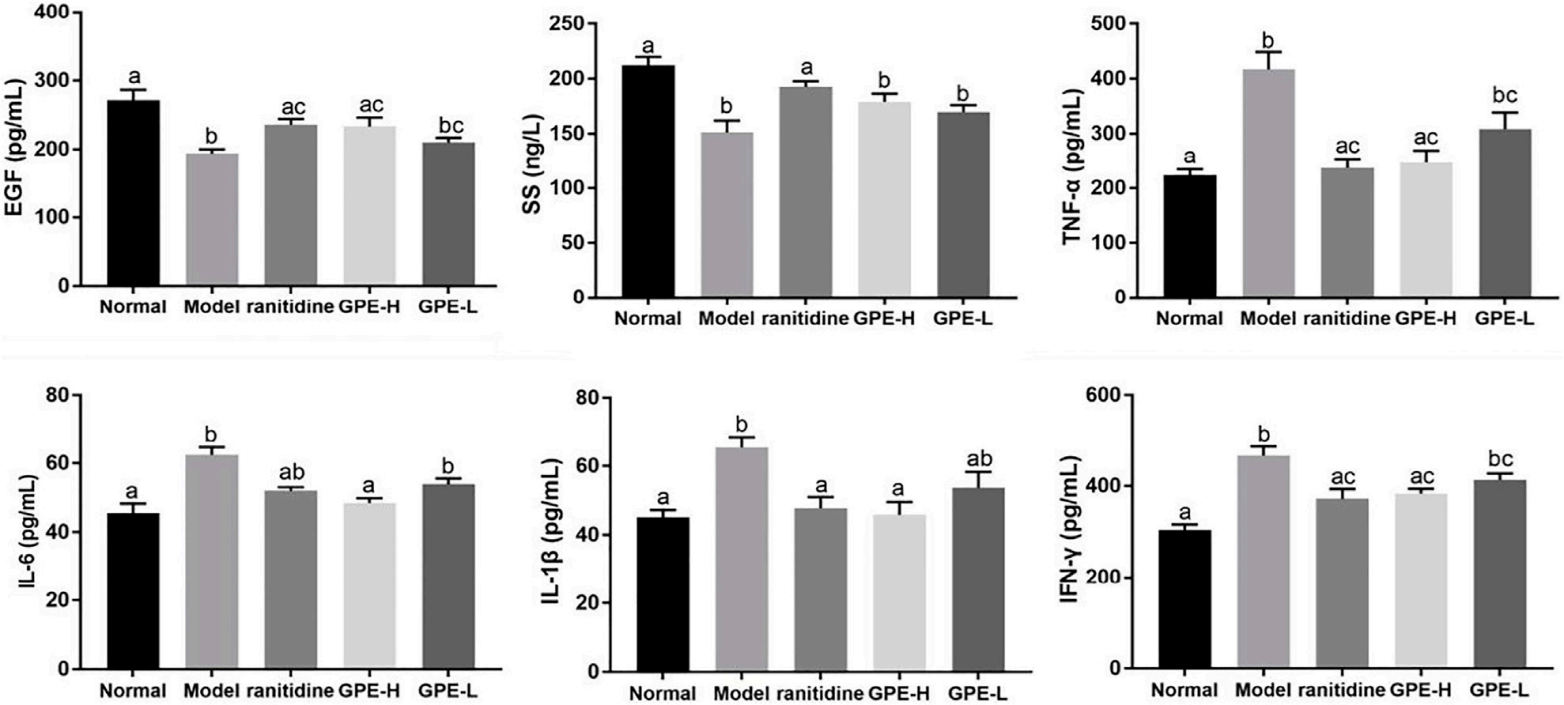
Serum levels of EGF, SS, TNF-α, IL-1β, IL-6, and IFN-γ in mice. The data are shown as mean ± SD (n = 8). ^a–c^ Mean values with different letters are significant difference (*p* < 0.05) according to analysis of variance.

As endogenous protective factors, serum EGF and SS levels were significantly reduced in the model group compared with those in normal group (*p* < 0.05; Figure 6). Compared with model mice, an increase of EGF induced by GPE-H and ranitidine were detected (*p* < 0.05). However, no significant change of SS level was observed between GPE group and model group.

TNF-α, IL-6, IFN-γ and IL-1β are common proinflammatory cytokines, and their levels could indicate the degree of inflammatory response (Strober and Fuss, 2011; Long et al., 2019). Compared with those in healthy mice, the levels of the inflammatory cytokines TNF-α, IL-6, IL-1β and IFN-γ were evidently increased in mice with gastric injury induced by ethanol/hydrochloric acid (*p* < 0.05; Figure 6), which showed that the mice were in an inflammatory state. After the mice were administered GPE-H and ranitidine by gavage, the decrease of inflammatory factor IL-6 and IL-1β was obvious compared with gastric injury model mice (*p* < 0.05), the effect was close to the normal group. A significant difference in TNF-α and IFN-γ levels was detected in GPE-H and ranitidine group in contrast to the model group (*p* < 0.05), and no significant difference compared with normal mice (*p* > 0.05). These results indicated that GPE-H and ranitidine efficiently alleviated inflammation.

### SOD1, SOD2, GSH-Px, CAT, γ-GCS, NF-κB, and IκBα mRNA Expression Levels in Gastric Tissue

**FIGURE 7:**
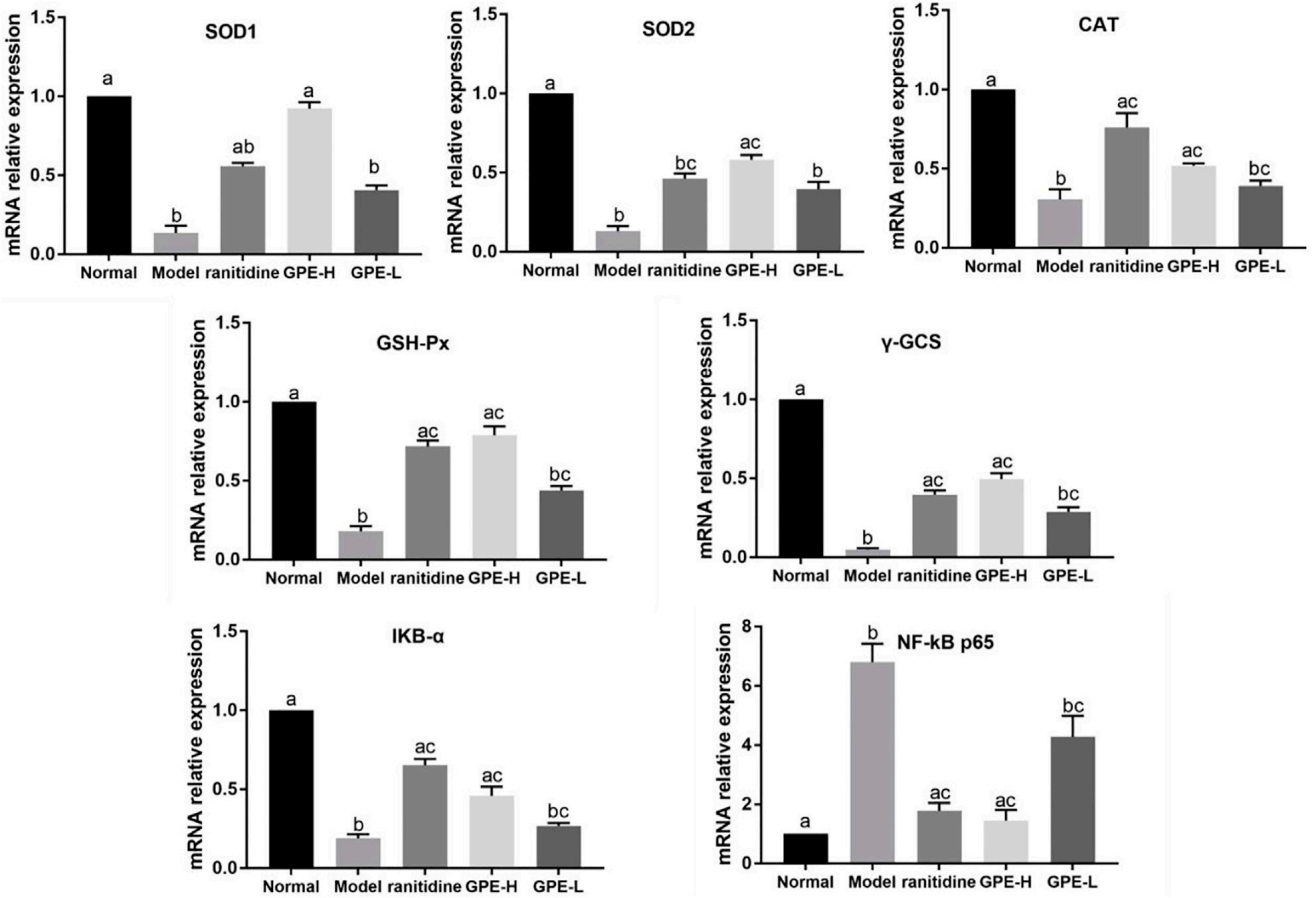
mRNA expression levels of SOD1*,* SOD2*,* CAT*,* γ-GCS*,* GSH-Px, NF-κB p65 and IκBα in gastric tissue of the different groups were investigated by qPCR. The data are shown as mean ± SD (n = 8). ^a–c^ Mean values with different letters are significant difference (*p* < 0.05) according to analysis of variance.

The mRNA expression of the antioxidant-related genes SOD1, SOD2, γ-GCS, GSH-Px, and CAT in gastric tissue was examined to explain the mechanism of GPE. The NF-κB pathway was also examined. The results showed that the mRNA expression levels of SOD1, SOD2, GSH-Px, CAT, γ-GCS and IκBα were significantly suppressed, and the mRNA expression of NF-κB was enhanced in the model mice compared with the normal mice (*p* < 0.05, Figure 7). GPE-H and ranitidine upregulated GSH-Px, CAT, γ-GCS and IκBα mRNA expression levels and simultaneously downregulated NF-κB mRNA expression levels at different degree compared with those in the model group (*p* < 0.05). Compared with the model group, ranitidine did not significantly increase the mRNA expression level of SOD (*p* > 0.05), but GPE-H had obvious effect on increasing that of SOD (*p* < 0.05), which was close to the normal group level. The results are consistent with the biochemical result.

## Discussion

At present, more than 60 kinds of flavonoid monomers have been identified in citrus. According to their structures, they can be divided into five categories: flavones, flavanones, flavonols, flavanols and anthocyanins (Gao et al., 2018). Flavanones are the most abundant flavonoids in citrus. The common aglycones are hesperitin and naringenin. They exist in the form of glycosides, mainly rutinoside and neohesperidin (Tripoli et al., 2007). Polymethoxy flavonoids are abundant in citrus fruits, and the most common flavonoids are nobiletin, tangeretin and sinensetin. They show good pharmacological activities (Tripoli et al., 2007). In GPE, the main flavanones were narirutin and hesperidin, and the polymethoxy flavonoids were nobiletin, tangeretin and 5-demethylnobiletin. In the nutrition survey, dietary flavonoid intake varies among people in different regions. The British adult’s dietary intake is 490 mg. The intake of dietary flavonoids of adults is 340 mg in US, which is low in China (Wang et al., 2020). Based on this study, 340 mg was selected as the reference for flavonoid intake. The equivalent dose ratio was obtained between humans and animals based on body surface area. The conversion factor between mouse (20 g) and human (70 kg) is 0.0026 (Nie et al., 2018). At the same time, the content of flavonoids in GPE was 16% by HPLC. The calculation process is as follows: 340 mg × 0.0026/0.02 kg/0.16 = 277 mg/kg. Based on the citrus peel extract was used in the literature (Ke et al., 2020), the high dose was finally determined to be 300 mg/kg and the low dose to be 150 mg/kg.

Ethanol, as an organic solvent, exerts strong corrosiveness on the gastric mucosa (Zhao et al., 2015). During ethanol metabolism *in vivo*, neutrophils release oxygen free radicals, which induce endothelial injury and microcirculatory disturbances, resulting in gastric mucosal injury (Alrashdi et al., 2012; Beiranvand and Bahramikia, 2020). As one of the components of human gastric juice, hydrochloric acid can promote the necrosis and abscission of gastric mucosa epithelium, and cause the rupture of vascular wall cells to accelerate gastric injury (Hamauzu et al., 2007). Pathological sections and morphological images of gastric tissue showed that the area of gastric injury and the shedding of gastric mucosal were reduced by GPE.

As one of the important protective factors of the gastrointestinal tract, EGF promotes the proliferation and differentiation of epithelial tissue and accelerates mucosal healing (Hui et al., 1993). SS could inhibit the secretion of gastric acid and pepsin, and promote the healing of gastric injury to protect the gastric mucosa (Pawlikowski and Melen-Mucha, 2003; Schubert, 2009). The results showed GPE had no significant effect on enhancing gastric mucosal defence factors.

Oxygen free radicals are one of the important mechanisms of ethanol-induced gastric injury (Tamura et al., 2013; Bhattacharyya et al., 2014). Excessive production of oxygen free radicals leads to lipid peroxidation. As the final metabolite of lipid peroxidation, MDA content indirectly reflects the degree of ROS attack on gastric tissue (Amirshahrokhi and Khalili, 2016). SOD, CAT and GSH-Px are important antioxidant enzymes that scavenge oxygen free radicals (Ozcan et al., 2013; Tan et al., 2018). GPE could improve the activities of SOD, CAT, and GSH-Px in gastric tissue and reduce MDA level, thus enhancing the antioxidant capacity. Also, studies have shown that stimulation of ethanol/hydrochloric acid can increase proinflammatory factors and trigger an inflammatory response (Wang et al., 2021). On the other hand, ROS promote the release of proinflammatory factors, thereby enhancing the intracellular signal cascade to exacerbate inflammation (Schoonbroodt and Piette, 2000; Reuter et al., 2010; Arulselvan et al., 2016). The results showed that GPE reduced the levels of the proinflammatory cytokines TNF-α, IL-6, IFN-γ and IL-1β to suppress the inflammatory response.

Oxidative stress is one of the important mechanisms of gastric tissue injury and is involved in many pathological processes (Bhattacharyya et al., 2014). Oxidative stress causes oxidative damage to biomolecules, leading to the release of damage-associated molecular patterns and cytokines (Gill et al., 2010). Cytokines can activate signaling pathways downstream of pattern-recognition receptors, such as NF-κB, and mitogen-activated protein kinase (MAPK) (Nakajima and Kitamura, 2013; Gunaseelan et al., 2017). It leads to increase the release of cytokines and chemokines, and then recruit and activate more inflammatory cells. Inflammation is closely related to oxidative stress, and NF-κB is the key link between them (Rodríguez-Ayala et al., 2005). When the body is under oxidative stress, the IκBα protein is degraded and activated to release NF-κB, further causing the production of proinflammatory factors (Lawrence, 2009; Mitchell and Carmody, 2018). Moreover, inflammatory factors, such as TNF-α, could induce the production of oxygen free radicals and promote the “oxidation burst” of neutrophils, to cause tissue damage (Pamir et al., 2009). Oxidative stress and inflammation influence each other by regulating transcription levels, forming a vicious circle. SOD, CAT, GSH-Px, glutathione are typical antioxidants. After the mice were treated with GPE, the mRNA expressions of SOD1, SOD2, CAT, GSH-Px and γ-GCS were enhanced. Furthermore, GPE suppressed the expression of NF-κB and elevated the expression of IκBα to inhibit inflammation, which may be related to its antioxidation abilities.

It is reported that hesperidin, a citrus flavonoid, protected against alcohol or acetic acid induced gastric injury by increasing the levels of SOD, glutathione, and CAT in the gastric mucosa. The protective effect is associated with the reduction of oxidative damage (Selmi et al., 2017; Silva et al., 2019). As the representative polymethoxyflavone compounds found in citrus peels, nobiletin and tangeretin could relieve ethanol-induced gastric injury by its antioxidant and anti-inflammatory activity (Li et al., 2017; Arafa and Islam., 2019). Nobiletin may inhibit the expression of inflammatory cytokines (TNF-α and IL-6) through the MAPK pathway to exert anti-inflammatory effects. Another type of polymethoxyflavone, 5-demethylnobiletin also showed good antioxidant and anti-inflammatory activities in other models (Wang et al., 2018; Wang et al., 2019). Although physiological activity of narirutin in a gastric injury model has not been investigated. But narirutin could restore anti-oxidation capability and inhibit the increase of NF-κB, TNF-α, IL-1β and other pro-inflammatory factors in the ethanol-induced liver injury (Park et al., 2013). Therefore, flavonoids in GPE may work cooperatively to possess anti-oxidant and anti-inflammatory effects, thereby alleviating ethanol/HCl-induced gastric damage. However, the main flavonoids in citrus peel, such as hesperidin and polymethoxyflavones, are poor in water solubility, which limited their bioavailability. Also, this property may also limit the clinical application of citrus peel flavonoids.

## Conclusion

In summary, GPE mainly contains the flavonoids narirutin, hesperidin, nobiletin, tangeretin and 5-demethylnobiletin. GPE has a protective effect against gastric injury induced by ethanol/hydrochloric acid by increasing antioxidant activity and inhibiting the inflammatory response. The qPCR results revealed that GPE enhanced the expression of antioxidant genes, which explained the possible mechanisms. This current study showed that GPE protected gastric tissue from ethanol/hydrochloric acid.

But improvement of bioavailability and the in-depth mechanism of GPE need to be further studied.

## Data Availability

The original contributions presented in the study are included in the article/supplementary material, further inquiries can be directed to the corresponding authors.

## Abbreviations

GPE, Gonggan peel extract; HPLC, high performance liquid chromatography; SOD, superoxide dismutase; GSH-Px, glutathione peroxidase; CAT, catalase; MDA, malondialdehyde; EGF, epidermal growth factor; SS, somatostatin; TNF-α, tumour necrosis factor-α; IFN-γ, interferon-γ; IL-1β, interleukin-1β; IL-6, interleukin-6; SOD1, copper/zinc superoxide dismutase; SOD2, manganese superoxide dismutase; γ-GCS, γ-glutamylcysteine synthetase; NF-κB, nuclear factor kappa-B; IκBα, inhibitor of NF-κB alpha; GAPDH, glyceraldehyde-3-phosphate dehydrogenase; ROS, reactive oxygen species; DPPH, 2,2′-diphenyl-1-picrylhydrazyl; ABTS, 2, 2′-azino-bis (3-ethylbenzothiazoline-6-sulfonic acid).

## References

[B1] Alrashdi A. S. Salama S. M. Alkiyumi S. S. Abdulla M. A. Hadi A. H. Abdelwahab S. I. (2012). Mechanisms of Gastroprotective Effects of Ethanolic Leaf Extract of *Jasminum Sambac* against HCl/Ethanol-Induced Gastric Mucosal Injury in Rats. Evid. Based Complement. Alternat Med. 2012, 786426. 10.1155/2012/786426 22550543 PMC3329065

[B2] Amirshahrokhi K. Khalili A. R. (2016). Gastroprotective Effect of 2-Mercaptoethane Sulfonate against Acute Gastric Mucosal Damage Induced by Ethanol. Int. Immunopharmacol 34, 183–188. 10.1016/j.intimp.2016.03.006 26967742

[B3] Amirshahrokhi K. Khalili A. R. (2015). The Effect of Thalidomide on Ethanol-Induced Gastric Mucosal Damage in Mice: Involvement of Inflammatory Cytokines and Nitric Oxide. Chem. Biol. Interact 225, 63–69. 10.1016/j.cbi.2014.11.019 25478868

[B4] Arafa E. S. A. Islam M. W. (2019). Tangeretin Protects against EthanolInduced Injury in Gastric Mucosa of Rats via its Antioxidants and Antiinflammatory Activity. FASEB 33, 505. 10.1096/fasebj.2019.33.1_supplement.505.16

[B5] Arulselvan P. Fard M. T. Tan W. S. Gothai S. Fakurazi S. Norhaizan M. E. (2016). Role of Antioxidants and Natural Products in Inflammation. Oxid Med. Cel Longev 2016, 5276130. 10.1155/2016/5276130 PMC5075620 27803762

[B6] Asikin Y. Taira I. Inafuku-Teramoto S. Sumi H. Ohta H. Takara K. (2012). The Composition of Volatile Aroma Components, Flavanones, and Polymethoxylated Flavones in Shiikuwasha (*Citrus Depressa* Hayata) Peels of Different Cultivation Lines. J. Agric. Food Chem. 60, 7973–7980. 10.1021/jf301848s 22804782

[B7] Beiranvand M. Bahramikia S. (2020). Ameliorating and Protective Effects Mesalazine on Ethanol-Induced Gastric Ulcers in Experimental Rats. Eur. J. Pharmacol. 888, 173573. 10.1016/j.ejphar.2020.173573 32956646

[B8] Bhattacharyya A. Chattopadhyay R. Mitra S. Crowe S. E. (2014). Oxidative Stress: An Essential Factor in the Pathogenesis of Gastrointestinal Mucosal Diseases. Physiol. Rev. 94, 329–354. 10.1152/physrev.00040.2012 24692350 PMC4044300

[B9] Cai X. T. Zhong J. W. Wen S. Y. Zhang X. Y. Liu Y. F. Ma L. K. (2021). Research Progress on Comprehensive Utilization of Tribute Citrus. J. Food Saf. Qual. 12, 3550–3556. 10.19812/j.cnki.jfsq11-5956/ts.2021.09.018

[B10] Chen X. M. Tait A. R. Kitts D. D. (2017). Flavonoid Composition of Orange Peel and its Association with Antioxidant and Anti-inflammatory Activities. Food Chem. 218, 15–21. 10.1016/j.foodchem.2016.09.016 27719891

[B11] Cirmi S. Ferlazzo N. Lombardo G. E. Ventura-Spagnolo E. Gangemi S. Calapai G. (2016). Neurodegenerative Diseases: Might Citrus Flavonoids Play a Protective Role. Molecules 21, 1312. 10.3390/molecules21101312 PMC6274333 27706034

[B12] da Silva L. M. Pezzini B. C. Somensi L. B. Bolda Mariano L. N. Mariott M. Boeing T. (2019). Hesperidin, a Citrus Flavanone Glycoside, Accelerates the Gastric Healing Process of Acetic Acid-Induced Ulcer in Rats. Chem. Biol. Interact 308, 45–50. 10.1016/j.cbi.2019.05.011 31095933

[B13] Deng H. M. Chao M. (2013). Studies on Extraction of Flavonoids from Tribute orange Peels and its Antibacterial Activities. The Food Industry 34, 21–23.

[B14] Erkan Ozcan M. Gulec M. Ozerol E. Polat R. Akyol O. (2004). Antioxidant Enzyme Activities and Oxidative Stress in Affective Disorders. Int. Clin. Psychopharmacol. 19, 89–95. 10.1097/00004850-200403000-00006 15076017

[B15] Gabriele M. Frassinetti S. Caltavuturo L. Montero L. Dinelli G. Longo V. (2017). Citrus Bergamia Powder: Antioxidant, Antimicrobial and Anti-Inflammatory Properties. J. Funct. Foods 31, 255–265. 10.1016/j.jff.2017.02.007

[B16] Gao Z. Gao W. Zeng S.-L. Li P. Liu E.-H. (2018). Chemical Structures, Bioactivities and Molecular Mechanisms of Citrus Polymethoxyflavones. J. Funct. Foods 40, 498–509. 10.1016/j.jff.2017.11.036

[B17] Gill R. Tsung A. Billiar T. (2010). Linking Oxidative Stress to Inflammation: Toll-Like Receptors. Free Radic. Biol. Med. 48, 1121–1132. 10.1016/j.freeradbiomed.2010.01.006 20083193 PMC3423196

[B18] Gunaseelan S. Balupillai A. Govindasamy K. Ramasamy K. Muthusamy G. Shanmugam M. (2017). Linalool Prevents Oxidative Stress Activated Protein Kinases in Single UVB-Exposed Human Skin Cells. PLoS One 12, e0176699. 10.1371/journal.pone.0176699 28467450 PMC5415184

[B19] Hamauzu Y. Forest F. Hiramatsu K. Sugimoto M. (2007). Effect of Pear (*Pyrus Communis L.*) Procyanidins on Gastric Lesions Induced by HCl/ethanol in Rats. Food Chem. 100, 255–263. 10.1016/j.foodchem.2005.09.050

[B20] Hui W. M. Chen B. W. Kung A. W. Cho C. H. Luk C. T. Lam S. K. (1993). Effect of Epidermal Growth Factor on Gastric Blood Flow in Rats: Possible Role in Mucosal protection. Gastroenterology 104, 1605–1610. 10.1016/0016-5085(93)90635-p 8500716

[B21] Kavoosi G. Amirghofran Z. (2017). Chemical Composition, Radical Scavenging and Anti-oxidant Capacity of Ocimum Basilicum Essential Oil. J. Essent. Oil Res. 29, 189–199. 10.1080/10412905.2016.1213667

[B22] Ke Z. Zhao Y. Tan S. Chen H. Li Y. Zhou Z. (2020). Citrus Reticulata Blanco Peel Extract Ameliorates Hepatic Steatosis, Oxidative Stress and Inflammation in HF and MCD Diet-Induced NASH C57BL/6 J Mice. J. Nutr. Biochem. 83, 108426. 10.1016/j.jnutbio.2020.108426 32559586

[B23] Ke Z. pan Y. Xu X. Nie C. Zhou Z. (2015). Citrus Flavonoids and Human Cancers. Jfnr 3, 341–351. 10.12691/jfnr-3-5-9

[B24] Lawrence T. (2009). The Nuclear Factor NF-kappaB Pathway in Inflammation. Cold Spring Harb Perspect. Biol. 1, a001651. 10.1101/cshperspect.a001651 20457564 PMC2882124

[B25] Ledesma-Escobar C. A. Luque de Castro M. D. (2014). Towards a Comprehensive Exploitation of Citrus. Trends Food Sci. Technol. 39, 63–75. 10.1016/j.tifs.2014.07.002

[B26] Li W. Wang X. Zhi W. Zhang H. He Z. Wang Y. (2017). The Gastroprotective Effect of Nobiletin against Ethanol-Induced Acute Gastric Lesions in Mice: Impact on Oxidative Stress and Inflammation. Immunopharmacol Immunotoxicol 39, 354–363. 10.1080/08923973.2017.1379088 28948855

[B27] Liu B. Feng X. Zhang J. Wei Y. Zhao X. (2019). Preventive Effect of Anji White Tea Flavonoids on Alcohol-Induced Gastric Injury through Their Antioxidant Effects in Kunming Mice. Biomolecules 9, 137. 10.3390/biom9040137 PMC6523235 30987336

[B28] Liu Y. Heying E. Tanumihardjo S. A. (2012). History, Global Distribution, and Nutritional Importance of Citrus Fruits. Compr. Rev. Food Sci. Food 11, 530–545. 10.1111/j.1541-4337.2012.00201.x

[B29] Long X. Zhao X. Wang W. Zhang Y. Wang H. Liu X. (2019). Protective Effect of Silkworm Pupa Oil on Hydrochloric Acid/Ethanol-Induced Gastric Ulcers. J. Sci. Food Agric. 99, 2974–2986. 10.1002/jsfa.9511 30479041

[B30] Mitchell J. P. Carmody R. J. (2018). NF-κB and the Transcriptional Control of Inflammation. Int. Rev. Cel Mol Biol 335, 41–84. 10.1016/bs.ircmb.2017.07.007 29305014

[B31] Nakajima S. Kitamura M. (2013). Bidirectional Regulation of NF-Κb by Reactive Oxygen Species: a Role of Unfolded Protein Response. Free Radic. Biol. Med. 65, 162–174. 10.1016/j.freeradbiomed.2013.06.020 23792277

[B32] Nie C. Zhao Z. Y. Xu X. D. Zhou Z. Q. (2017). Separation and Purification of Total Flavonoids from Lane Late Navel orange by AB-8 Macroporous Resins. Sci. Technol. Food Industry 38, 221–225. 10.13386/j.issn1002-0306.2017.18.042

[B33] Nie H. Mei Z. Wang R. Zhao B. Gao Y. Chen J. (2018). Bushen Recipe and its Disassembled Prescriptions Inhibit Inflammation of Liver Injury Associated with Concanavalin A through Toll-Like Receptor 3/9 Signaling Pathway. Mol. Med. Rep. 18, 1682–1691. 10.3892/mmr.2018.9082 29845244

[B34] Pamir N. McMillen T. S. Kaiyala K. J. Schwartz M. W. LeBoeuf R. C. (2009). Receptors for Tumor Necrosis Factor-Alpha Play a Protective Role against Obesity and Alter Adipose Tissue Macrophage Status. Endocrinology 150, 4124–4134. 10.1210/en.2009-0137 19477937 PMC2736076

[B35] Park H. Y. Ha S. K. Eom H. Choi I. (2013). Narirutin Fraction from Citrus Peels Attenuates Alcoholic Liver Disease in Mice. Food Chem. Toxicol. 55, 637–644. 10.1016/j.fct.2013.01.060 23416143

[B36] Pawlikowski M. Melen-Mucha G. (2003). Perspectives of New Potential Therapeutic Applications of Somatostatin Analogs. Neuro Endocrinol. Lett. 24, 21–27. 12743527

[B37] Qin S. Yin J. Huang S. Lin J. Fang Z. Zhou Y. (2019). Astragaloside IV Protects Ethanol-Induced Gastric Mucosal Injury by Preventing Mitochondrial Oxidative Stress and the Activation of Mitochondrial Pathway Apoptosis in Rats. Front. Pharmacol. 10, 894. 10.3389/fphar.2019.00894 31474858 PMC6704233

[B38] Raish M. Ahmad A. Ansari M. A. Alkharfy K. M. Aljenoobi F. I. Jan B. L. (2018). Momordica Charantia Polysaccharides Ameliorate Oxidative Stress, Inflammation, and Apoptosis in Ethanol-Induced Gastritis in Mucosa through NF-kB Signaling Pathway Inhibition. Int. J. Biol. Macromol 111, 193–199. 10.1016/j.ijbiomac.2018.01.008 29307809

[B39] Reuter S. Gupta S. C. Chaturvedi M. M. Aggarwal B. B. (2010). Oxidative Stress, Inflammation, and Cancer: How Are They Linked? Free Radic. Biol. Med. 49, 1603–1616. 10.1016/j.freeradbiomed.2010.09.006 20840865 PMC2990475

[B40] Rodríguez-Ayala E. Anderstam B. Suliman M. E. Seeberger A. Heimbürger O. Lindholm B. (2005). Enhanced RAGE-Mediated NFkappaB Stimulation in Inflamed Hemodialysis Patients. Atherosclerosis 180, 333–340. 10.1016/j.atherosclerosis.2004.12.007 15910860

[B41] Schoonbroodt S. Piette J. (2000). Oxidative Stress Interference with the Nuclear Factor-Kappa B Activation Pathways. Biochem. Pharmacol. 60, 1075–1083. 10.1016/s0006-2952(00)00371-3 11007944

[B42] Schubert M. L. (2009). Gastric Exocrine and Endocrine Secretion. Curr. Opin. Gastroenterol. 25, 529–536. 10.1097/MOG.0b013e328331b62a 19726980

[B43] Selmi S. Rtibi K. Grami D. Sebai H. Marzouki L. (2017). Protective Effects of orange (*Citrus Sinensis L.*) Peel Aqueous Extract and Hesperidin on Oxidative Stress and Peptic Ulcer Induced by Alcohol in Rat. Lipids Health Dis. 16, 152. 10.1186/s12944-017-0546-y 28806973 PMC5556677

[B44] Sharma K. Mahato N. Lee Y. R. (2019). Extraction, Characterization and Biological Activity of Citrus Flavonoids. Rev. Chem. Eng. 35, 265–284. 10.1515/revce-2017-0027

[B45] Strober W. Fuss I. J. (2011). Proinflammatory Cytokines in the Pathogenesis of Inflammatory Bowel Diseases. Gastroenterology 140, 1756–1767. 10.1053/j.gastro.2011.02.016 21530742 PMC3773507

[B46] Suo H. Zhao X. Qian Y. Sun P. Zhu K. Li J. (2016). *Lactobacillus Fermentum* Suo Attenuates HCl/Ethanol Induced Gastric Injury in Mice through its Antioxidant Effects. Nutrients 8, 155. 10.3390/nu8030155 26978395 PMC4808883

[B47] Tamura M. Matsui H. Kaneko T. Hyodo I. (2013). Alcohol Is an Oxidative Stressor for Gastric Epithelial Cells: Detection of Superoxide in Living Cells. J. Clin. Biochem. Nutr. 53, 75–80. 10.3164/jcbn.13-32 24062603 PMC3774929

[B48] Tan B. L. Norhaizan M. E. Liew W. P. Sulaiman Rahman H. (2018). Antioxidant and Oxidative Stress: A Mutual Interplay in Age-Related Diseases. Front. Pharmacol. 9, 1162. 10.3389/fphar.2018.01162 30405405 PMC6204759

[B49] Toth P. P. Patti A. M. Nikolic D. Giglio R. V. Castellino G. Biancucci T. (2016). Bergamot Reduces Plasma Lipids, Atherogenic Small Dense LDL, and Subclinical Atherosclerosis in Subjects with Moderate Hypercholesterolemia: A 6 Months Prospective Study. Front. Pharmacol. 6, 299. 10.3389/fphar.2015.00299 26779019 PMC4702027

[B50] Tripoli E. Guardia M. L. Giammanco S. Majo D. D. Giammanco M. (2007). Citrus Flavonoids: Molecular Structure, Biological Activity and Nutritional Properties: A Review. Food Chem. 104, 466–479. 10.1016/j.foodchem.2006.11.054

[B51] Wang M. Meng D. Zhang P. Wang X. Du G. Brennan C. (2018). Antioxidant protection of Nobiletin, 5-Demethylnobiletin, Tangeretin, and 5-Demethyltangeretin from Citrus Peel in Saccharomyces Cerevisiae. J. Agric. Food Chem. 66, 3155–3160. 10.1021/acs.jafc.8b00509 29526093

[B52] Wang R. Sun F. Ren C. Zhai L. Xiong R. Yang Y. (2021). Hunan Insect Tea Polyphenols Provide Protection Against Gastric Injury Induced by HCl/Ethanol through an Antioxidant Mechanism in Mice. Food Funct. 12, 747–760. 10.1039/d0fo02677h 33367402

[B53] Wang Y. Zang W. Ji S. Cao J. Sun C. (2019). Three Polymethoxyflavones Purified from Ougan (Citrus Reticulata Cv. Suavissima) Inhibited LPS-Induced NO Elevation in the Neuroglia BV-2 Cell Line via the JAK2/STAT3 Pathway. Nutrients 11, 791. 10.3390/nu11040791 PMC6521056 30959824

[B54] Wang Y. Lin X. C. He S. Q. Tian C. Li L. Guo X. L. (2020). Estimated Dietary Polyphenol Intake in Chinese Adults: The CHNS Study. Acta Medicinae Universitatis Scientiae et Technologiae Huazhong 49, 556–561. 10.3870/j.issn.1672-0741.2020.05.008

[B55] World Health Organization (2019). Global Status Report on Alcohol and Health 2018[M]. Geneva: World Health Organization.

[B56] Zhang Y. Xiu M. Jiang J. He J. Li D. Liang S. (2006). Novokinin Inhibits Gastric Acid Secretion and Protects against Alcohol-Induced Gastric Injury in Rats. Alcohol 56, 1–8. 10.1016/j.alcohol.2016.08.003 27814789

[B57] Zhao X. Wang Q. Qian Y. Song J. L. (2013). Ilex Kudingcha C.J. Tseng (Kudingcha) Prevents HCl/Ethanol-Induced Gastric Injury in Sprague-Dawley Rats. Mol. Med. Rep. 7, 1613–1616. 10.3892/mmr.2013.1402 23546392

[B58] Zhao Z. Gong S. Wang S. Ma C. (2015). Effect and Mechanism of Evodiamine against Ethanol-Induced Gastric Ulcer in Mice by Suppressing Rho/NF-Кb Pathway. Int. Immunopharmacol 28, 588–595. 10.1016/j.intimp.2015.07.030 26225926

[B59] Zou Z. Xi W. Hu Y. Nie C. Zhou Z. (2016). Antioxidant Activity of Citrus Fruits. Food Chem. 196, 885–896. 10.1016/j.foodchem.2015.09.072 26593569

